# KIF4A drives gliomas growth by transcriptional repression of Rac1/Cdc42 to induce cytoskeletal remodeling in glioma cells

**DOI:** 10.7150/jca.77238

**Published:** 2022-11-21

**Authors:** Hui Zhang, Seng Meng, Kun Chu, Sufang Chu, Yue-Chao Fan, Jin Bai, Zheng-Quan Yu

**Affiliations:** 1 Department of Neurosurgery, the first Affiliated Hospital of Soochow University, Suzhou, Jiangsu, China.; 2 Cancer Institute, Xuzhou Medical University, Xuzhou, Jiangsu, China.; 3 Center of Clinical Oncology, the Affiliated Hospital of Xuzhou Medical University, Xuzhou, Jiangsu, China.; 4 Department of Neurosurgery, the Affiliated Hospital of Xuzhou Medical University, Xuzhou, Jiangsu, China.

**Keywords:** Glioma, KIF4A, proliferation, metastasis, morphology, cytoskeletal

## Abstract

Glioma is one of the most prevalent cancers diseases in the worldwide. Kinesin superfamily protein 4 (KIF4), a KIF member classified in Kinesin 4 has been indicated as a mediator acted in tumorigenesis of human cancer. However, the mechanism of KIF4A on glioma is yet to be investigated. This study aimed to explore the potential function and mechanism of KIF4A in gliomas. We analyzed the KIF4A expression and the prognosis in gliomas patients using The Cancer Genome Atlas (TCGA) databases. KIF4A level in normal human astrocyte cell (NHA) and glioma cell lines were examined by Western blot. We studied the function of KIF4A on proliferation, migration, invasion, cell cycle in glioma cell lines using a series of *in vitro* and* in vivo* experiments. Chromatin Immunoprecipitation (ChIP) analysis was applied to searching potential KIF4A related downstream in glioma. We identified the significant up-regulated expression of KIF4A both in glioma tissues and cell. Glioma patients with elevated KIF4A expression have shorter survival. Down-regulation of KIF4A exerted inhibitory effect on cell proliferation, invasion and migration. We crucially identified that KIF4A drives gliomas growth by transcriptional repression of Rac1/Cdc42 to induce cytoskeletal remodeling in glioma cells. Knockdown of KIF4A decreased RohA, Rac1, Cdc42, Pak1 and Pak2 expression level. Our study provided a prospect that KIF4A functions as an oncogene in glioma.

## Introduction

Gliomas are the most prevalent primary malignant tumors of the brain and spinal cord tumors. They can occur anywhere in the central nervous system (CNS), but mainly occur in the brain and glial tissue 1. Malignant gliomas have distinctive features such as rapid proliferation, invasion of peripheral CNS tissue and abnormal vascularization 2. Due to this pathological features, its higher malignancy results in a worse prognosis for the patient 3. With the large improvement and application of sequencing, glioma characterization has been revealed strong relationships with specific epigenetic modifications, transcriptome alterations, and clinical manifestations to define subtypes 4, 5. Our understanding of cancer biology has evolved considerably with the advent of cancer genetics and molecular characterization. Histologically, they have the characteristics of normal glial cells and are often named according to these similarities 6. However, search for pathological features biomarker that caused the development and progression of gliomas is still a subject of research.

Kinesin superfamily protein 4 (KIF4), a KIF member classified in Kinesin 4, localizes in the nucleoplasm. KIF4A have been identified as KIF4 homolog and works as a mitotic motor implicated in chromosome segregation during mitosis 7. Furthermore, depletion of KIF4A in human cells results in defective chromosome condensation and segregation, specific performances: disorder of cell cycle and instability of chromosomes 8. KIF4A also prolongs the survival of neurons by targeting PARP1 which is involved in recovery from DNA damage. The knockdown of KIF4A in nude mice promotes malignant transformation from normal cell and tumor formation. Actually, it has been proved that KIF4A plays an important role in various tumors, such as oral cancer, liver cancer, lung cancer, colorectal carcinoma, breast cancer and prostate cancer 9. However, little is known about the correlation between KIF4A and gliomas. Recent studies demonstrated that KIF4A interacts with BRCA2 by the KIF4A C-terminal cargo binding domain and BRCA2 C-terminal conserved region when exposed to laser micro-irradiation 10. In progression of oral squamous carcinomas, KIF4A controls cellular proliferation via spindle assembly checkpoint activation 11. However, the association between KIF4A expression and glioma have not been reported, and the role of KIF4A in glioma was never investigated.

The cytoskeleton is a complex network of protein filaments that shapes cells. It is composed of microfilaments, intermediate filaments and microtubules, and extends from the cell membrane to nucleus 12. During the cell cycle, cytoskeleton promotes the separation of chromosomes and mitosis. About morphology, normal cell of the same type maintains a uniform shape based on the tight connection of filaments, some cells undergo malignant transformation and appear as an array of shapes and sizes. Finally, in cancer cell migration cytoskeleton forms specialized structures, such as flagella, cilia, and lamellipodia which are believed to be the actual motor to pull the cell move forward. The molecular mechanisms underlying the cytoskeleton involve Rho GTPase family which is typified by Rho, Rac and Cdc42 13. They have been shown to regulate actin dynamics as molecular switches 14. KIF4A also known as microtubule-based motor protein which moves across the microtubule, transporting cellular cargo within the cell. Microtubules are polymers of tubulin that form part of cytoskeleton 15. There are two main functions: transportation of chromosomes and regulation of microtubule dynamics 16. In other words, the protein KIF4A is in contact with cytoskeleton and may have mutual effects. This is our initial motivation which lead to a more in-depth and specific research.

In this study, considering the carcinogenicity of KIF4A in certain human tumors, we investigate the potential connection related to glioma. We queried the expression of KIF4A in TCGA database and found that it is enriched in glioma. We showed that KIF4A is highly expressed in glioma and indicated poor prognosis. We also showed that KIF4A promotes glioma cells growth *in vitro* and *in vivo*. KIF4A regulates cell mobility through regulating F-actin cytoskeleton organization. In addition, we found that KIF4A regulates F-actin cytoskeleton organization through activating Rac1 and Cdc42 at transcription level. It suggested KIF4A may regulate glioma cells growth and mobility through the Rac1/Cdc42 pathway.

## Materials and Methods

### Bioinformatical analyses

The Cancer Genome Atlas (TCGA) databases were used to analyzed KIF4A expression in glioma and normal tissue. Clinicopathological features including overall survival (OS) and disease-free survival (DFS) was evaluated linked to glioma patients KIF4A expression. The KIF4A expression data and clinical data of patients with gliomas (including low-grade glioma and glioblastoma) and normal brain tissues in the TCGA database were obtained from UCSC Xena (http://xena.ucsc.edu/) and GTEx database (http://commonfund.nih.gov/GTEx/). The transcriptome data from TCGA and GTEx (163 glioma tissues and 207 normal brain tissues) were merged for further analysis by online bioinformatics tool The Gene Expression Profiling Interactive Analysis (GEPIA).

### Cell Culture

Normal human astrocyte cell and glioma cells were purchased from cell bank of Chinese Academy of Sciences. NHA, T98G, A172, LN229, U87 and U251 were correspondingly cultured in RMPI-1640 or DMEM Medium, supplemented with 10% FBS, 100 U/mL each penicillin and streptomycin separately. Cell incubated condition is 37 °C humidified with 5% CO2.

### Cell Transfection

Small interfering RNA (SI) specific for KIF4A (siKIF4A) and non-specific control (NC) were purchased from (Gene-Pharma, Shanghai, China) and transfected by siLentFect Lipid Reagent (Bio-Rad Laboratories, Inc.) according to the manufacturer's protocol when glioma cells were grown to above 50% confluency. Six hours after transfection, the medium containing transfection reagents was replaced by fresh medium. The siRNAs sequences were described as follows: S1 sense: GGUCCAGACUACUACUCUATT; S2 sense: GGAAUGAGGUUGUGAUCUUTT; NC sense: UUCUCCGAACGUGUCACGUTT.

### Stable cell line generation

For stable suppression KIF4A expression, KIF4A short hairpin RNA (SH) expression and non-specific control lentivirus (NC) were obtained from Gene-Pharma. U87 and U251 cells were infected with lentivirus for 48 h, and then were selected with 2 ng/ml puromycin for 2 weeks, with the medium refreshed every 3 days. The shRNA target sequences were described as follows: SH sense: GGAATGAGGTTGTGATCTT; NC sense: TTCTCCGAACGTGTCACGT.

### RNA isolate, RT-PCR and qPCR

We prepared total RNA by using TRIzol Reagent (Invitrogen), synthesized cDNA with HiScript Q RT SuperMix for qPCR Kit (Vazyme Biotech). Quantitative realtime PCR was performed on ABI-7500 using UltraSYBR Mixture Kit (CWBIO Biotech). The primers using for qPCR analysis were listed as followed: 5'-CTGCAATTGGTTGGCGTCTC-3' (forward) and 5'-CAGCGCCACTCTTACAGGAA-3' (reverse) for KIF4A; 5'-GTAACCCGTTGAACCCCATT-3' (forward) and 5'-CCATCCAATCGGTAGTAGCG-3' (reverse) for 18S.

### Cell proliferation and colony formation assay

CCK-8 (Beyotime, China) was used to measure cell proliferative capacity. 96-well plates planted with transfected cells (5×10^3^ cells/group) were incubated at 37 °C with 5% CO_2_ for 24h, 48h, 72h. 10µl of CCK-8 solution was added to each cell well at each time point and incubate for 2h subsequently. The absorbance at 450 nm of each group was tested by the microplate reader. For colony formation assay, 7 × 10^2^ cells were cultured in six-well plate at 37 °C for 14 days, visible colonies were washed twice with PBS, fixed, and stained with 4% paraformaldehyde and crystal violet, respectively. The number of colonies was counted visually.

### Transwell Assays

Firstly, U87 or U251 cells were planted into the upper wells of chambers (BD Biosciences, USA) coated with or without Matrigel (BD Biosciences), then, cultured in a 200 μL FBS-free RMPI-1640 or DMEM medium. The lower wells of the chambers containing 390 μL culture medium an 10 μL FBS. 24h later, removed the medium of the upper wells, and fixed cells with 100% methanol and then stained with 0.1% crystal violet for 15 min. The stained cells were photographed using an Olympus microscope.

### Cell cycle analysis

The U251 and U87 glioma cells were transfected with siRNA. After 48 h, the medium was replaced with a medium without FBS. On the next day, the cell was rinsed with PBS and incubated in a fresh medium containing FBS for 0, 3, and 6 h. The cells were fixed with 70% ethanol at 4 °C overnight. On the next day, the cells were stained with propidium iodide and RNase A. Afterward, the samples were analyzed using a FACS Canto flow cytometer (BD Biosciences). The cell distribution in the different phases of the cell cycle was analyzed using the ModFit LT3.0 software.

### Western Blot

The total content of cellular protein harvested from glioma cells were extracted using RIPA lysis buffer (Keygen, Nanjing, China) and the protein amounts was determined using a BCA kit (Keygen, Nanjing, China). SDS-PAGE separated same volumes proteins of each lane, then blotted onto PVDF membranes. Primary antibodies were incubated overnight at 4 °C, and HRP-labeled secondary antibody (ABclonal, 1:5,000) 2h at room temperature after blocked with 5% BSA. Signal of immunoreactivities were photographed by ECL reagent (NCM Bio, China) on Tanon 5200 automatic chemiluminescence imaging system (Tanon, China).

### Chromatin immunoprecipitation assay

ChIP assay was performed according to the protocol of ChIP assay kit (Upstate Biotechnology, Lake Placid, NY). U87 and U251 cells cultured in 100 mm dish (about 1 × 10^7^) were cross-linked by adding formaldehyde to final concentration of 1% and incubated in room temperature for 10 min, washed twice with cold PBS containing protease inhibitors, lysed by ChIP lysis buffer, sonicated to shear DNA at 4 °C to reduce the average length. Sonicated lysates were then diluted 10-fold with ChIP dilution buffer and reduced the non-specific binding with protein A-agarose for 1 h at 4 °C, in this step, 20 μl of lysate were taken out as input control, then followed by incubation with anti-KIF4A or anti-IgG (as negative control) at 4 °C overnight with rotation. After reversal washes with a series of buffers, qRT-PCR was performed to amplify the genomic region of the Rac1 and Cdc42 flanking the potential KIF4Abinding sites.

### Tumor xenograft study

All animal experiments were carried out in accordance with institutional guidelines and regulations of the Animal Care and Use Committee and Ethics Committee of Xuzhou Medical University approved all animal experiments. Male BALB/c nude mice were purchased from the (Vital River Laboratory Animal Technology. China) and randomly divided into two groups (SH group and NC group) and 6 mice per group. Glioma cell line U87 were counted and then re-suspended to 1 × 10^5^ cells/μL using PBS. Then intracranially injected with cell suspension by using a micro-syringe at about 0.5 cm from the posterior right side of junction between the anterior midline and outer canthus. The needle was inserted by approximately 0.5 cm. Mice were observed after injection to monitor the tumor size by bioluminescence images after 6 weeks (Night OWL II LB983; Berthold Technologies). Hematoxylin-eosin staining was accorded to the description of the HE staining kit (BBI Life Sciences; Number: E607318).

### Statistical Analysis

All collected data were analyzed using Statistical Product and Service Solutions (SPSS 23.0, IBM, USA) and presented in form of mean ± standard deviation. Student's t-test were used to tested the differences between two groups. Comparison among three groups was analyzed using One-way ANOVA test. The association between KIF4A staining and the clinicopathological factors of the patients was evaluated using the χ^2^ test. Survival analysis was implemented using the Kaplan-Meier method. *p*<0.05 suggested statistically significant differences.

## Results

### KIF4A overexpression occurs in glioma and associates with advanced grade glioma

**Figure 1:**
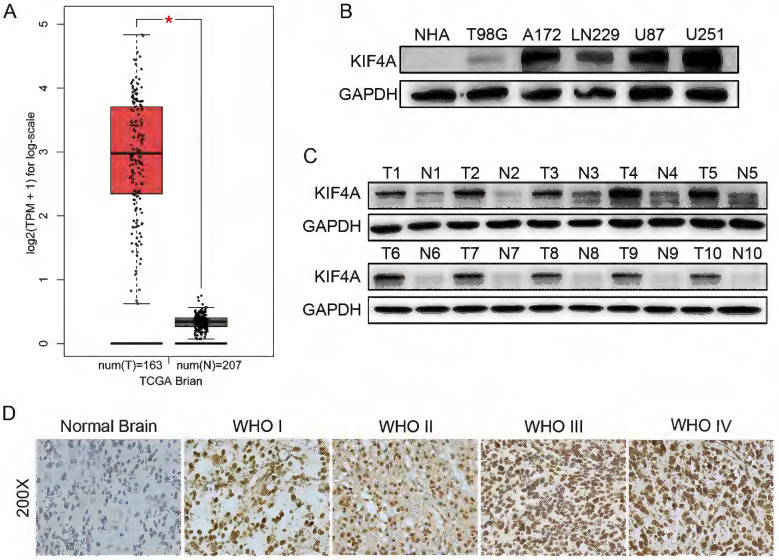
** Expression of KIF4A is upregulated in glioma tissues and glioma cell lines. (A)** Expression level of KIF4A in glioma tissues (T=163) and normal tissues (N=207) analyzed in TGCA database (* *p*<0.05). **(B)** Expression patterns of KIF4A in glioma cell lines and NHA cell line detected by western blot. **(C)** Expression patterns of KIF4A in glioma tissues (T) and normal tissues (N) detected by western blot. **(D)** KIF4A immunostaining in TMAs are shown.

To reveal the role of KIF4A in glioma, we analyzed the expression of KIF4A in TCGA database, results showed that KIF4A expressions were significantly overexpressed in brain tumor samples than normal brain tissues (*p* < 0.05 Fig. 1A). Further, KIF4A protein expressions were evaluated in normal human astrocyte cell (NHA) and several glioma cells, the western blot assays revealed that KIF4A protein levels were upregulated in glioma cells compared with that in NHA cell (Fig. 1B). Western blot also showed that KIF4A was overexpressed in glioma tissue samples (T) compared with the adjacent normal brain tissues (N) (Fig. 1C). KIF4A expression was then examined in an expanded brain cancer cohort by a tissue microarray (TMA). Our tissue assay results showed that KIF4A expression was upregulated in advanced grade WHO III/IV compared with grade WHO I/II in brain cancer tissues (Fig. 1D, Table 1).

### KIF4A overexpression indicates poor glioma prognosis

**Figure 2:**
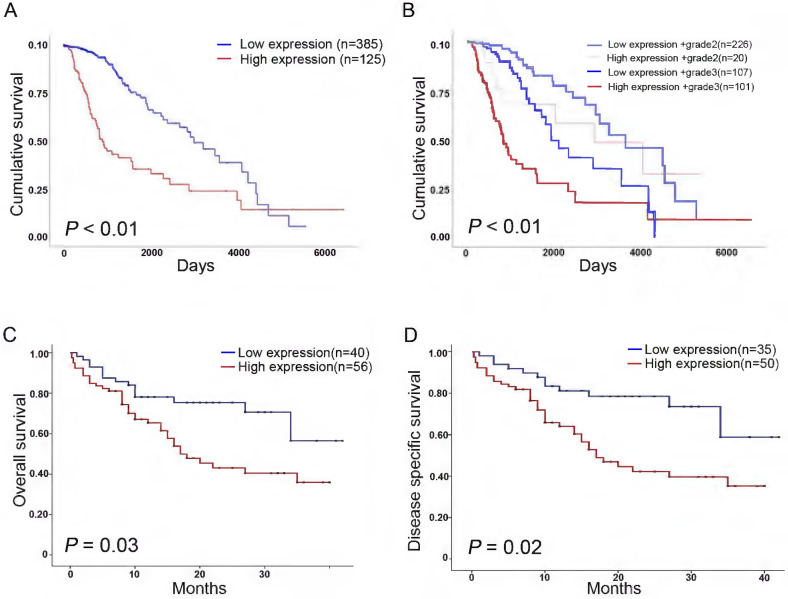
** Expression of KIF4A negatively associated with survival rate in glioma patients. (A)** High KIF4A expression is associated with poorer overall cumulative survival for glioma patients (p<0.01). **(B)** High KIF4A expression relevant to poorer cumulative survival for glioma patients in grade3 than grade2 (p<0.01). **(C)** OS rate of glioma patients with high or low expression of KIF4A (p=0.03). **(D)** DFS rate of glioma patients with high or low expression of KIF4A (p=0.02). Analyzed using the Kaplan-Meier analyses and log-rank test.

We analyzed the association of KIF4A expression with survival in patients with glioma using online database. Survival analysis of TCGA datasets revealed that high KIF4A expression was significantly correlated with poor cumulative survival (*p* < 0.01, Fig. 2A), and high KIF4A expression in different grade glioma showed poor survival compared with low KIF4A expression (*p* < 0.01, Fig. 2B). We also analyzed the association of KIF4A with survival in another glioma cohort, data showed that high KIF4A expression was significantly correlated with poor overall survival (OS) (*p* = 0.03, Fig. 2C) and disease-free survival (DFS) (*p* = 0.02, Fig. 2D).

### Knockdown of KIF4A represses glioma cells proliferation

**Figure 3:**
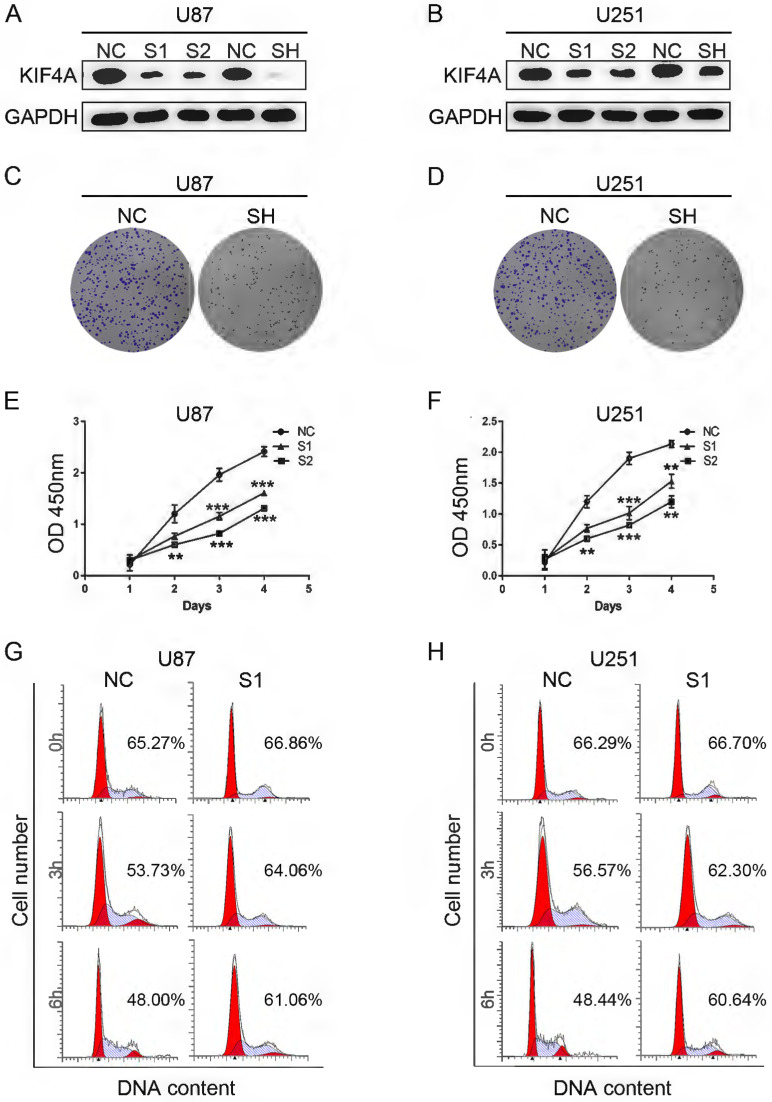
** Knockdown of KIF4A suppresses glioma cell proliferation and cell cycle *in vitro*. (A, B)** KIF4A expression at the protein level in U87 and U251 glioma cells was evaluated by Western blot after transfection. **(C, D)** KIF4A knockdown inhibits the capacity of colony formation in U251 and U87 glioma cells compared with negative transfected cells (NC). **(E, F)** CCK-8 assays revealed that silence of KIF4A suppresses (SH) cell proliferation of U251 and U87 glioma cells compared with negative transfected cells (NC). **(E, F)** Knockdown of KIF4A increased G0/G1 phase cell population, as detected by flow cytometric analysis following Annexin FITC and PI staining. Data are shown as mean ± SD. * p<0.05; ** p<0.01; *** p<0.001.

To investigate the biofunction role of KIF4A in glioma cells, siRNAs and shRNA targeting KIF4A was used to knockdown KIF4A expression. Western blots showed that both the siRNAs and shRNA could effectively repress KIF4A expression in U87 and U251 cells (Fig. 3A and 3B). Colony formation assays also suggested that KIF4A knockdown could effectively repress glioma cells growth (Fig. 3C and 3D). CCK-8 assays revealed that KIF4A knockdown could significantly reduce cell proliferation in U87 and U251 cells (Fig. 3E and 3F). Given that cell proliferation is controlled by the cell cycle, we investigated if the proliferation of DKC1 knockdown cells was due to the change in cell cycle. After cycle synchronization, transfected and control cells were incubated in the fresh medium for 0, 3, and 6 h. The flow cytometer data showed that the G1 population has a slower rate of decline in the two cell lines with DKC1 knockdown compared with the control cells (Fig. 3G and 3H).

### Knockdown of KIF4A represses glioma cells migration and invasion

**Figure 4:**
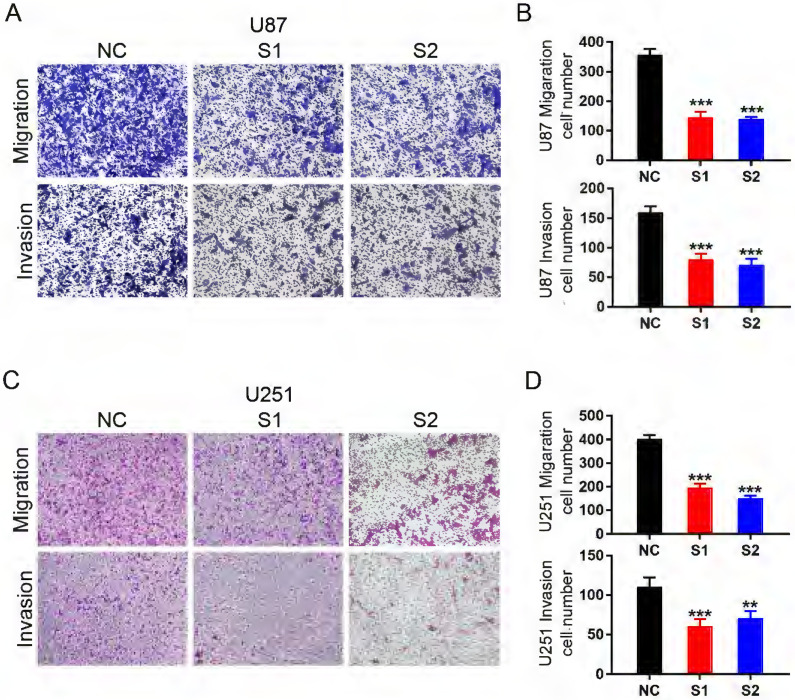
** Knockdown of KIF4A inhibits glioma cell migration and invasion. (A, C)** Representative pictures of migration and invasion in U87 and U251 cells with KIF4A knockdown (S1, S2) and controls (NC). **(B, D)** Number of cell migration and invasion per field were counted in five random fields for KIF4A knockdown and control groups. Data are shown as mean ± SD. * p<0.05; ** p<0.01; *** p<0.001.

In malignant gliomas, especially glioblastoma, most patients immediately die of distant tumor metastasis. Thus, we investigated the role of KIF4A in glioma migration and invasion by transwell assays. KIF4A Knockdown significantly repressed cell migration and invasion in U87 and U251 cells (Fig. 4A-4D). It revealed a positive role of KIF4A in promoting cell migration and invasion in glioma cells.

### Silencing KIF4A affected the morphology of glioma cells

**Figure 5:**
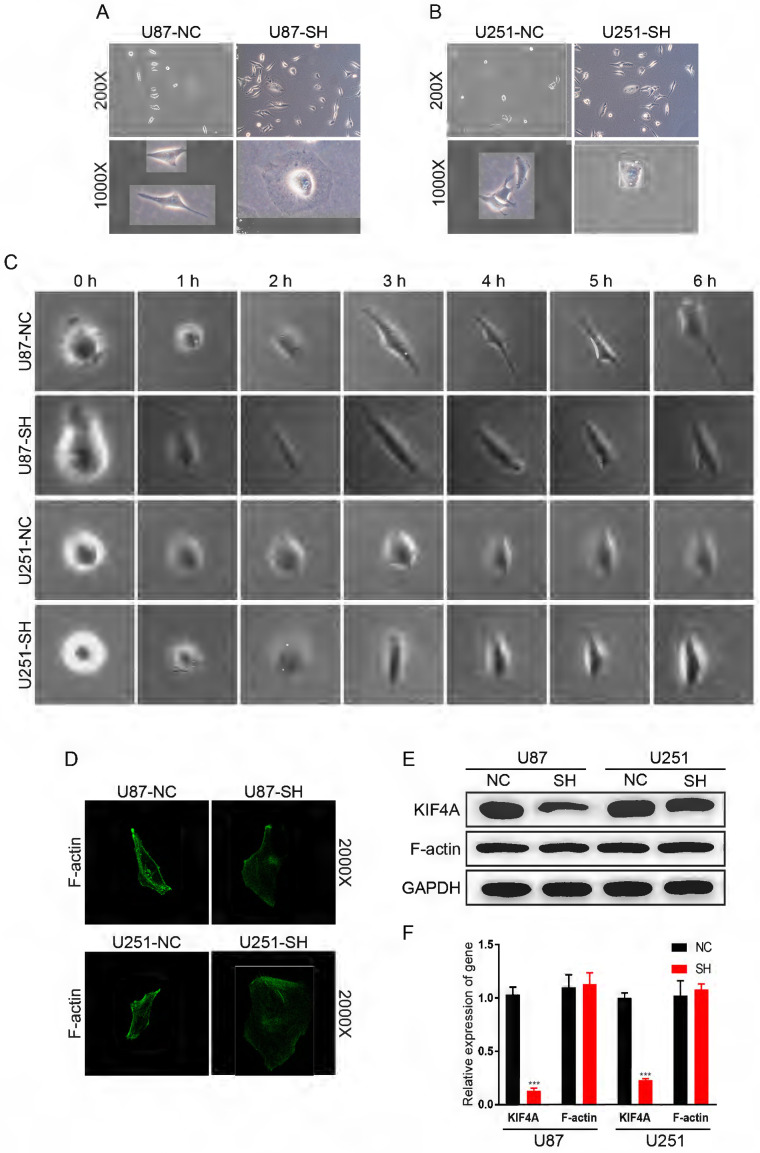
** Silencing KIF4A affected the morphology of glioma cells. (A, B)** Representative pictures of morphological changes of U87 and U251 cells with KIF4A knockdown (SH) compared with controls (NC). **(C)** The images effect of knockdown of KIF4A on the morphological changes of glioma cells was observed in each one hour. **(D)** Immunofluorescent staining confirmed that knockdown KIF4A could retain the location of F-actin. **(E)** Western blot analysis of the relative protein levels of F-actin in KIF4A knockdown, and control groups of U87 and U251 cells. GAPDH was used as a reference control. Data are shown as mean ± SD. * p<0.05; ** p<0.01; *** p<0.001.

Interestingly, we observed that silencing KIF4A can retain cells morphology, a change from spindle-shaped to oval-shaped (Fig. 5A and 5B). We refined the observation time point to further observed the effect of KIF4A on the morphological progression of glioma cells in each one hour, and obtained the same conclusion (Fig. 5C). It suggested that silencing KIF4A may induce cytoskeletal remodeling to regulate glioma cell mobility. Immunofluorescent staining confirmed that knockdown KIF4A could retain the location of F-actin (Fig. 5D). However, western blot assays showed that silencing KIF4A did not affect the expression of F-actin protein level (Fig. 5E and 5F).

### KIF4A transcriptionally regulates Rac1 and Cdc42

**Figure 6:**
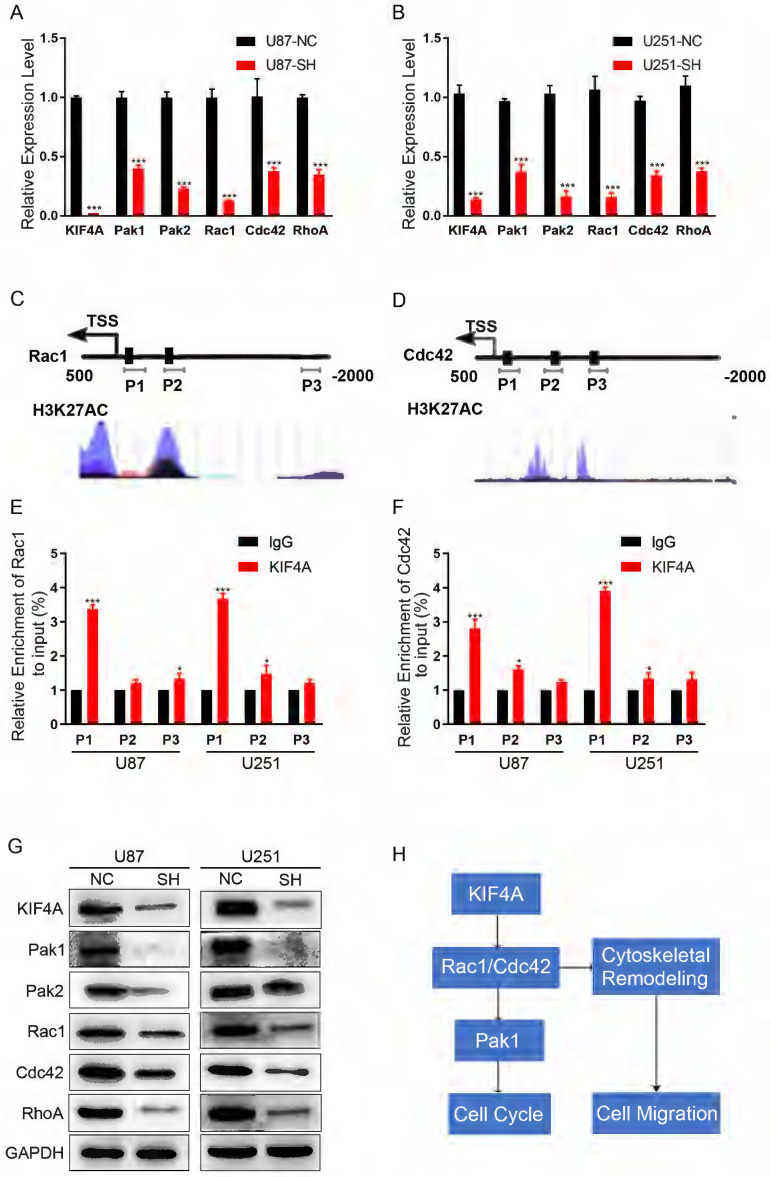
** Silencing of DKC1 alters the related signaling molecules in U87 and U251 cells. (A, B)** Expression patterns of KIF4A related signaling molecules in U87 and U251 cell lines detected by qRT-PCR.** (C, D)** Full sequence of Rac1 and Cdc42 promoter. P1, P2, P3 shows the regions of promoter detected by the paired primers. **(E, F)** ChIP-qPCR analysis of KIF4A binding at P1, P2, p3 loci. **(G)** Western blot analysis of the relative protein levels of Pak1, Pak2, Rac1, Cdc42, and RhoA in KIF4A knockdown, and control groups of U87and U251 cells. GAPDH was used as a reference control. **(H)** Figure abstract about the potential signal pathway that KIF4A may participated and affected. Data are shown as mean ± SD. * p<0.05; ** p<0.01; *** p<0.001.

Actin cytoskeleton dynamics is regulated by small GTPases of the Rho family, RhoA and Rac1/Cdc42 govern cell motility cycle 17, 18. As we have found that silencing KIF4A induced F-actin cytoskeleton organization, we hypnosis that whether KIF4A regulated F-actin through RhoA or Rac1/Cdc42. We calculated the effects of silencing KIF4A on their mRNA expressions by using qRT-PCR, Result showed that silencing KIF4A significantly reduced RhoA, Rac1 and Cdc42 mRNA level, as well as their target genes Pak1 and Pak2 (Fig. 6A and 6B). Furthermore, the promoters of Rac1 and Cdc42 have a strong signaling of H3K27Ac (Fig. 6C and 6D), H3K27Ac is associated with the higher activation of transcription and therefore defined as an active enhancer mark. ChIP assays showed that KIF4A can interact with the promoter of Rac1 and Cdc42 (Fig. 6E and 6F), it suggested that KIF4A can promote Rac1 and Cdc42 expression at the transcription level. Western blots also showed that silencing KIF4A repress the protein levels of RohA, Rac1, Cdc42, Pak1 and Pak2 (Fig. 6G).

### Knockdown of KIF4A inhibited glioma cells growth *in vivo*

**Figure 7:**
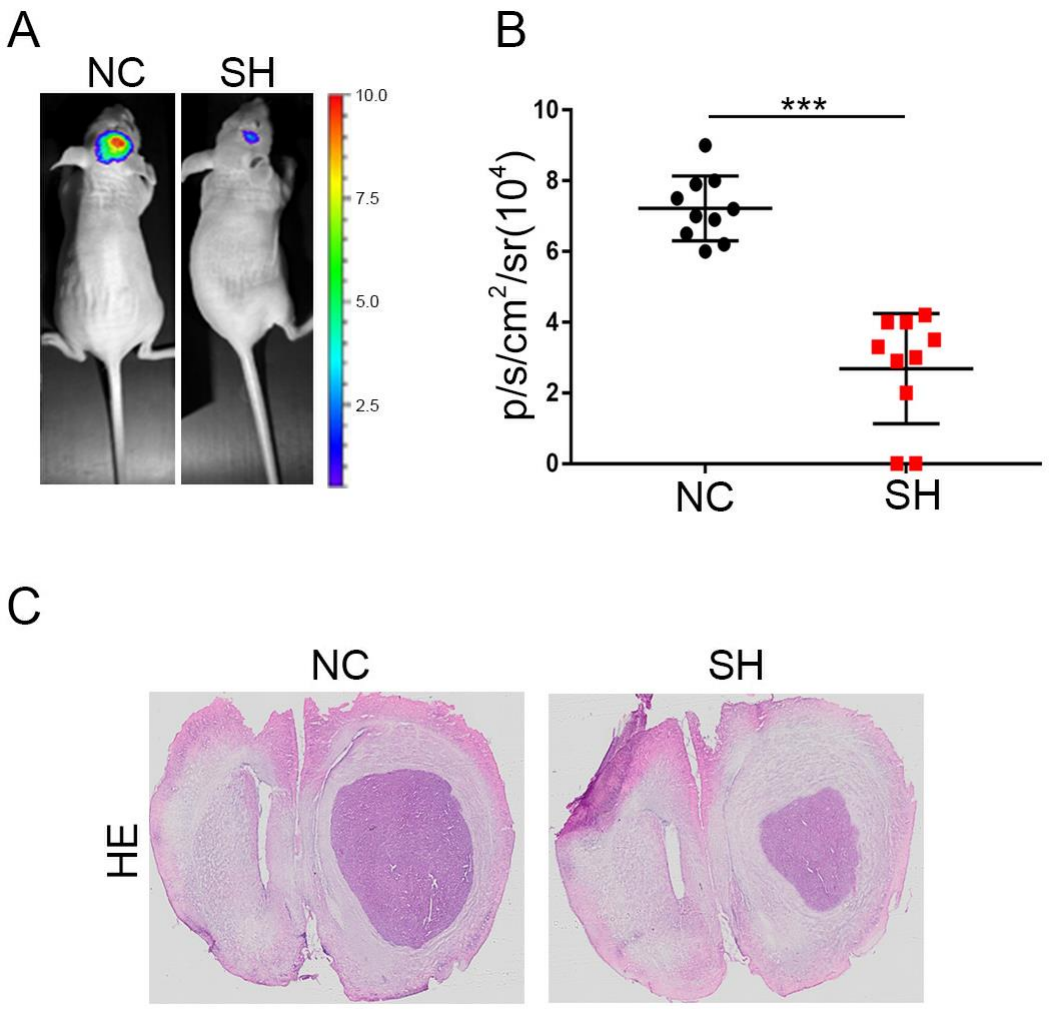
** Knockdown of KIF4A restrains the tumor formation of glioma cells *in vivo*. (A, B)** Representative bioluminescence images and statistical analysis of tumor growth in mice via *in situ* implantation of indicated cells in mice brain. **(C)** The tumor sections were performed immunochemistry HE staining, representative images were shown. Data are shown as mean ± SD. * p<0.05; ** p<0.01; *** p<0.001.

The effects of KIF4A on the proliferation of glioma cells were validated in a xenograft mouse model injected with the same number of SH or NC glioma cells *in situ* implantation in the brain of nude mice, and data showed that knockdown of KIF4A inhibited glioma cells growth (Fig. 7A and 7B). Furthermore, HE staining for tissue sections from SH and NC tumors was performed. The representative images showed that knockdown of KIF4A resulted in smaller tumor size in the excised mice brain tissue (Fig. 7C). Taken together, our study reveals KIF4A transcriptionally actives Rac1/Cdc42 pathway to regulate cell cycle and cytoskeleton remodeling to govern cell migration and proliferation (Fig. 6H).

## Discussion

Kinesin proteins make up a large superfamily of molecular motors, known as kinesin superfamily proteins (KIFs) 19. Intracellular organelle trafficking is critical for cell morphogenesis and function in the form of independent microtubules and motor proteins 20. Kinesins and kinesin-related proteins make up a large superfamily of molecular motors that transport cargoes such as vesicles, organelles, protein complexes, and mRNAs in a microtubule-dependent and ATP-dependent manner in neuronal and non-neuronal cells 21. Consider the role of KIF in these processes, dysregulation of KIFs expression and function would result in cell death, abnormality, and tumorigenesis. Several studies have demonstrated that altered KIF proteins play a critical role in the progression of multiple human cancers 22, 23. As a critical member of the KIF family, KIF4A has been reported to be highly expressed and play a role in the progression of various cancers 24, 25. However, the expression and function of KIF4A in glioma cells have never been investigated.

Brain tumors are generally classified using the World Health Organization (WHO) system that is largely based on pathological features. Grade I and II tumors are considered non-malignant, and Grade III and IV tumors are malignant, Grade IV tumors also termed glioblastoma 26. Gliomas are the most malignant and aggressive form of brain tumors, and account for the majority of brain cancer related deaths. Malignant gliomas, including glioblastoma are treated with radiation and temozolomide, with only a minor benefit in survival time 27. With the current standard of medical treatment, consisting of maximal safe resection followed by external beam radiation and concomitant temozolomide (TMZ) with then maintenance chemotherapy, median overall survival (OS) for patients with newly diagnosed GBM is only 12-18 months 28. A number of advances have been made in understanding glioma biology, pathways involved in tumor growth, apoptosis, invasion, and angiogenesis provide rational targets in glioma 29. There is an aim to individualize treatment based on the specific molecular abnormalities of a particular tumor.

Rho GTPases regulate cytoskeletal and cell adhesion dynamics and thereby coordinate a wide range of cellular processes, including cell migration, cell polarity and cell cycle progression 30. Alterations in Rho GTPase signal pathway contribute to malignant transformation, neurological abnormalities and immunological diseases 31. Activation of RhoA and Rac1, 2 members of the family, has been shown to induce the formation of membrane protrusions and retractions as well as the regulation of actin polymerisation into filaments 32. The small GTPase Rac1 has been implicated in a variety of dynamic cell biological processes, including cell proliferation, cell survival, cell-cell contacts, epithelial mesenchymal transition (EMT), cell motility, and invasiveness 33. As regards the p21-activated kinases (PAKs), they act as downstream effectors of Cdc42 and Rac1 GTPases. All PAKs are characterized by an N-terminal p21-GTPase binding domain and a highly conserved C-terminal kinase domain 34. These proteins exert a critical role in many cellular processes, including cell morphology, survival, transcription, cell cycle progression, motility, apoptosis 35. PAK1 might also be related to resistance to chemotherapies. Accordingly, PAK1 overexpression partially overcomes the 5-fluoro-uracile (5-FU) -induced growth inhibition of human colon cancer cells xenografted in -SCID mice, whereas PAK1 inhibition acts in synergy with 5-FU treatment 36. Such PAK1-induced resistance to chemotherapies might be related to the involvement of PAK1 in DNA damage repair 37, 38.

In the present research, KIF4A have been proved overexpressed in glioma cells according to the TCGA analysis of clinical specimens, higher KIF4A expression level was correlated with poor cumulative survival, and high KIF4A expression in different grade glioma showed poor survival compared with low KIF4A expression, which is key finding of prognostic significance of KIF4A for glioma patients.

Our work is the initial demonstration of KIF4A biologic function correlated with malignant progress of glioma cells. A series of assays *in vitro* and *in vivo* revealed that KIF4A down-regulation inhibited tumor growth and reduced cell migration and invasion. Flow cytometry assays showed that knockdown KIF4A inhibited cell cycle in glioma cells.

Interestingly, we observed that silencing KIF4A can retain cells morphology and changed cytoskeleton organization, while KIF4A has no effect on F-actin protein level. To precisely unravel the pathways that were related to the function of KIF4A in cytoskeletal remodeling of glioma, we hypnosis that whether KIF4A regulated F-actin through RhoA or Rac1/Cdc42. qRT-PCR analysis was conducted and results showed that silencing KIF4A significantly reduced RhoA, Rac1 and Cdc42 mRNA level, as well as their target genes Pak1 and Pak2.

Then we confirmed that the promoters of Rac1 and Cdc42 have a strong signaling of H3K27Ac, which is associated with the higher activation of transcription and therefore defined as an active enhancer mark. Our ChIP assays showed that KIF4A can interact with the promoter of Rac1 and Cdc42, it suggested that KIF4A can promote Rac1 and Cdc42 expression at the transcription level. Western blots also showed that silencing KIF4A repress the protein levels of RohA, Rac1, Cdc42, Pak1 and Pak2. These findings indicate that KIF4A plays an oncogenic role in glioma development and is potential prognostic marker for gliomas patients.

## Conclusions

In conclusion, our study shows in the first time that aberrant expression of KIF4A contributes to the proliferation and cytoskeletal remodeling progression of glioma cells. Knockdown KIF4A decreased glioma cell proliferation and metastasis. These findings shed light on the prospects for KIF4A as a potential biomarker and target in prognosis and therapy for gliomas.

## Figures and Tables

**Table 1:** KIF4A staining and clinicopathological characteristics of 380 glioma patients

Variables	KIF4A staining			
	Low (%)	High (%)	Total	*P* *
All cases	181 (47.6)	199 (52.4)	380	
**Age**				
≤42 years	80(47.9)	87(52.1)	167	0.925
>42 years	101(47.4)	112(52.6)	213	
**Gender**				
Male	98(46.7)	112(53.3)	210	0.676
Female	83(48.8)	87(51.2)	170	
**WHO grade**				
Benign (I-II)	127(60.0)	85(40.0)	212	<0.001
Malignant (III-IV)	54(32.1)	114(67.9)	168	
**Histological type**				
Astrocytoma	45(49.5)	46(50.5)	91	0.510
Glioblastoma	9(20.9)	34(79.1)	43	
Oligodendroglioma	3(30.0)	7(70.0)	10	
Ependymoma	1(33.3)	2(66.7)	3	
Pilocyticastrocytoma	2(25.0)	6(75.0)	8	

* Two-sided Fisher's exact tests. Some cases were not available for the information.

## Abbreviations

KIF4: Kinesin superfamily protein 4
TCGA: The Cancer Genome Atlas
NHA: Human astrocyte cell
chip: Chromatin Immunoprecipitation
CNS: Central nervous system
OS: Overall survival
DFS: Disease-free survival
SI: Small interfering RNA
SH: Short hairpin RNA
qRT-PCR: Quantitative real-time polymerase chain reaction
CCK-8: Cell Counting Kit 8
ChIP: Chromatin immunoprecipitation
TMA: a tissue microarray
